# The Impact of a Single-Day Hands-On Dermatology Workshop on Medical Students’ Knowledge and Confidence

**DOI:** 10.7759/cureus.113329

**Published:** 2026-07-24

**Authors:** Sebastian M Beller, Diane S Hong, Karolina D Perez, Daniel C Butler, Mahdieh Fazel

**Affiliations:** 1 Dermatology, College of Medicine, University of Arizona, Tucson, USA

**Keywords:** academic dermatology, dermatology knowledge and education, dermatology perception, interactive workshop, medical school education

## Abstract

Dermatology is an essential component of undergraduate medical education. Limited exposure may leave medical students underprepared to recognize common skin conditions and may contribute to misconceptions regarding the clinical scope of dermatology. This study evaluated the educational impact of a brief dermatology workshop on medical students’ knowledge, diagnostic confidence, and perceptions of the specialty. A single-institution pre-post interventional study was conducted during “Dermatology Day,” a structured educational workshop for first-year medical students incorporating didactic instruction, hands-on learning, and patient interaction. Anonymous surveys were administered before and after the event. The pre-event survey collected demographic information and assessed baseline dermatology knowledge using a 10-question quiz and a lesion description task scored on a five-point rubric. Confidence in core dermatologic assessment skills and perceptions of dermatology were measured using Likert-scale items. The post-event survey repeated knowledge and confidence measures and included additional questions assessing shifts in specialty perceptions. Quantitative outcomes were analyzed using paired t-tests, and free-text responses underwent thematic analysis.

A total of 27 students completed the pre-event survey, and 16 completed the post-event survey. Knowledge quiz scores did not significantly change following the intervention. However, a non-significant trend toward improvement was observed in task-based performance, with higher post-event lesion description accuracy scores. Significant gains were observed across multiple confidence domains, including identifying key dermatologic history questions, observing and describing skin findings, understanding the steps of a full skin examination, and recognizing indications for and techniques of skin biopsy. Students also reported improved understanding of dermatologists’ clinical roles and greater clarity regarding the range of conditions managed within the specialty. Awareness of diagnostic challenges in skin of color increased significantly. Qualitative analysis of free-text responses identified the following two primary themes: the educational value of interacting with patients with chronic dermatologic conditions and the benefit of directly observing skin lesions during the workshop. Students frequently reported that these experiences enhanced their understanding of dermatologic disease and broadened their perception of the specialty’s clinical breadth. Although differences in response rates between pre- and post-surveys and the absence of a control group limit causal interpretation, the consistent improvements in confidence and perception measures suggest an immediate educational benefit. These findings indicate that concise, hands-on educational interventions may meaningfully enhance confidence in dermatologic assessment skills and in understanding of the specialty among early medical learners. Integrating similar workshops into medical school curricula may represent an effective strategy to address the limited dermatology exposure present in many training programs. Future studies should assess long-term knowledge retention and evaluate the impact of repeated or expanded dermatology educational experiences.

## Introduction

Skin disease accounts for approximately one-fifth of all outpatient visits in the United States, representing a substantial burden on both patients and the healthcare system [1]. At the individual level, dermatologic illness significantly affects quality of life, contributes to healthcare expenditures, and frequently serves as an early manifestation of systemic disease [2]. Despite its broad clinical relevance, dermatology remains underrepresented in medical education. Currently, the mean number of hours allocated to dermatology instruction over the four years of medical school is reported to be only 12.6 (median: 10; range: 0-34), which typically does not include hands-on experiences or direct patient exposure [3]. This limited engagement leaves students underprepared to recognize common dermatologic presentations and may perpetuate misconceptions that dermatology is primarily cosmetic or tangential to general practice, rather than medically essential.

Recent education research has demonstrated the value of brief, interactive learning experiences in improving specialty knowledge and correcting misconceptions in other medical fields, such as internal medicine, nephrology, geriatrics, and surgery. For instance, brief nephrology “escape rooms” improved recall of key concepts and attitudes toward the specialty, while geriatrics “myths of aging” intervention improved attitudes toward older adults [3-8]. Interactive game-based and discussion-based learning has even shown superior results to traditional lecture-based instruction for students new to the operating room in surgical specialties [3,9]. However, few studies have examined the effectiveness of comparable interactive interventions within dermatology education. Given the limited curricular exposure to dermatology in most medical schools, such interventions may offer a valuable means of improving both competence and perceptions of the specialty [10-13].

To address these gaps, Dermatology Day (Derm Day) was developed as an educational event for first-year medical students consisting of didactic teaching, hands-on technique workshops, and opportunities to interact with patients with chronic skin conditions. The objective of this study was to evaluate the efficacy of this brief, structured dermatology intervention. Specifically, the primary objective was to assess changes in students' objective dermatologic knowledge and self-reported task-based confidence, while the secondary objective was to evaluate shifts in students' perceptions of the specialty and its clinical scope. By quantifying these prioritized primary and secondary outcomes, this study aimed to inform future curricular strategies and generate insights that support more robust integration of dermatology into early medical education curricula.

## Materials and methods

Study design

This study employed a pre-post interventional design to evaluate the impact of a single-day dermatology workshop, Dermatology Day (Derm Day), on prioritized primary outcomes (medical student knowledge and confidence across core dermatologic evaluation tasks) and secondary outcomes (perceptions of the specialty and its clinical scope). Surveys were administered before and after the intervention to assess changes across these domains.

Participants

The target population included first-year medical students (MS1s) enrolled in the College of Medicine at the University of Arizona during the 2025-2026 academic year. Participation was voluntary and open to all MS1 students. Inclusion criteria were enrollment as a first-year medical student and attendance at the Derm Day event. Students who declined participation or were not part of the MS1 class were excluded.

Intervention

Derm Day was a structured, interactive educational program designed to provide early exposure to clinical dermatology. The 2-h event consisted of a brief introductory overview of dermatology and session goals, a focused didactic lecture on lesion morphology and common diagnostic approaches, rotating hands-on stations for basic skin examination and biopsy/suturing techniques using suture kits, and facilitated discussion with volunteer patients living with chronic dermatologic conditions. All sessions were supervised by dermatology faculty, residents, and third or second-year medical student facilitators.

Data collection

Data were collected using anonymous electronic surveys administered via Qualtrics (Provo, UT: Qualtrics, LLC). The pre-event survey collected demographic and baseline data, including optional questions on race, ethnicity, and free response questions on prior dermatology exposure (e.g., shadowing, employment, or personal/family history of skin disease).

The survey assessed knowledge using 10 multiple-choice questions covering dermatologic morphology, diagnosis, and management of common conditions. Confidence was evaluated using Likert-scale items assessing students’ self-reported ability to perform a dermatologic history, accurately describe lesions, understand biopsy indications, and conduct a full skin examination. Perceptions were assessed using statements addressing understanding of dermatology’s scope, recognition of dermatologists’ role in managing serious disease, and attitudes toward the specialty.

The post-event survey repeated all knowledge and confidence questions to assess changes in learning outcomes. Additional perception questions evaluated shifts in specialty understanding (e.g., “After today’s event, I have a clear understanding of the types of conditions dermatologists treat”) and future interest in dermatology (e.g., “I am more likely to consider dermatology as a specialty after this event”). Two open-ended questions solicited qualitative feedback regarding the most valuable component of the workshop and suggestions for future improvements. The complete pre- and post-intervention survey instruments are provided in appendix.

Outcome measures

The primary outcomes were changes in knowledge and confidence-related scores from pre- to post-intervention. Secondary outcomes included shifts in students' perceptions and attitudes toward dermatology, such as interest in the specialty and understanding of its scope.

Statistical analysis

Quantitative survey data were analyzed using Welch’s t-tests to compare pre- and post-intervention scores. Because surveys were administered anonymously and responses were not linked at the individual level, pre- and post-intervention samples were treated as independent groups. Given unequal sample sizes and potential variance differences, Welch's t-test was selected. Continuous variables were summarized as means with standard deviations, while categorical variables were summarized as frequencies and percentages. Likert-scale responses were converted to numerical values for statistical analysis. Statistical significance was defined as p<0.05.

Knowledge scores were calculated as the percentage of correct responses on the multiple-choice assessment. Performance on the lesion description task was evaluated using a five-point rubric awarding one point each for correct identification of primary morphology, color, border characteristics, anatomical location, and secondary features such as scale. Confidence and perception measures were derived from Likert-scale items evaluating students’ comfort with dermatologic history-taking, lesion description, biopsy techniques, full skin examination steps, and understanding of dermatology’s clinical scope. Qualitative free-text responses were analyzed using inductive thematic analysis. Responses were reviewed and coded to identify recurring themes related to the perceived value of the workshop and suggestions for improvement.

## Results

A total of 27 students completed the pre-event survey, and 16 completed the post-event survey. Participant demographics are summarized in Table 1. Most participants reported no prior dermatology exposure (22/27, 81.5%), with only five of 27 students (18.5%) indicating previous experiences such as shadowing or clinical observation. Knowledge quiz scores remained similar between the pre- and post-intervention assessments (88.43%±18.32 vs. 86.72%±20.14; p=0.78). Similarly, lesion description accuracy improved modestly but did not reach statistical significance using Welch’s t-test (mean score: 2.63±1.71 pre-event vs. 3.56±1.55 post-event; p=0.075).

**Table 1:** Participant demographic characteristics (n=27). Values shown as n (%). Race and ethnicity were self-reported. Previous experiences in dermatology include any prior shadowing, work, or clinical exposure.

Demographics (n=27)	n (%)
Race	
White	14 (51.9)
Black or African American	2 (7.4)
Asian	7 (25.9)
Other	4 (14.8)
Ethnicity	
Hispanic	6 (22.2)
Non-Hispanic	21 (77.8)
Previous experiences in dermatology	
Yes	5 (18.5)
No	22 (81.5)

In contrast, significant improvements were observed across several confidence-related domains (Table 2). Students reported greater confidence in identifying key dermatologic history questions following the intervention (2.85±1.18 vs. 4.19±0.81; p<0.001). Comfort observing and describing skin findings also increased significantly (2.81±1.16 vs. 3.94±1.09; p=0.003). Students demonstrated improved understanding of the steps involved in performing a full skin examination (3.19±1.42 vs. 4.50±1.00; p=0.001) and greater confidence in understanding the indications and techniques for skin biopsy (3.00±1.12 vs. 4.38±0.60; p<0.001).

**Table 2:** Pre- and post-event quantitative outcomes for knowledge, task-based performance, confidence, and perception measures. *Statistically significant p-value. Values shown as mean±standard deviation. Likert-scale items were scored from 1 to 5, with higher values indicating greater confidence or understanding. P-values were calculated using Welch’s t-tests. MCQ: multiple choice questions

Domain	Scoring method	Measure	Pre-event (n=27)	Post-event (n=16)	p-Value
Knowledge assessment	10-item MCQ/matching; 1 point per correct response	Knowledge quiz score, mean %±SD	88.43±18.32	86.72±20.14	0.78
Task-based performance	1 point each for primary morphology, color, border, location, secondary features	Lesion description accuracy, mean points±SD	2.63±1.71	3.56±1.55	0.075
Confidence in dermatologic assessment	Likert 1-5 (1=strongly disagree → 5=strongly agree)	Confidence identifying key dermatologic history questions, mean points±SD	2.85±1.18	4.19±0.81	8.03571×10^-5^*
		Comfort observing and describing skin findings, mean points±SD	2.81±1.16	3.94±1.09	0.002971402*
		Understanding of full skin examination process, mean points±SD	3.19±1.42	4.50±1.00	0.001062616*
		Understanding of skin biopsy indications and techniques, mean points±SD	3.00±1.12	4.38±0.60	5.23477×10^-6^*
Perceptions of dermatology	Likert 1-5 (1=strongly disagree → 5=strongly agree)	Understanding of dermatologists' clinical role, mean points±SD	3.74±0.84	4.50±0.50	0.000613146*
		Clarity regarding conditions managed by dermatologists, mean points±SD	3.74±0.80	4.63±0.48	4.76378×10^-5^*
		Awareness of diagnostic challenges in skin of color, mean points±SD	4.04±0.79	4.50±0.50	0.024525908*

Perceptions of dermatology also shifted significantly following the workshop. Students reported greater understanding of dermatologists’ clinical role (3.74±0.84 vs. 4.50±0.50; p<0.001) and increased clarity regarding the range of conditions managed by dermatologists (3.74±0.80 vs. 4.63±0.48; p<0.001). Awareness of diagnostic challenges in skin of color also improved (4.04±0.79 vs. 4.50±0.50; p=0.025).

**Figure 1:**
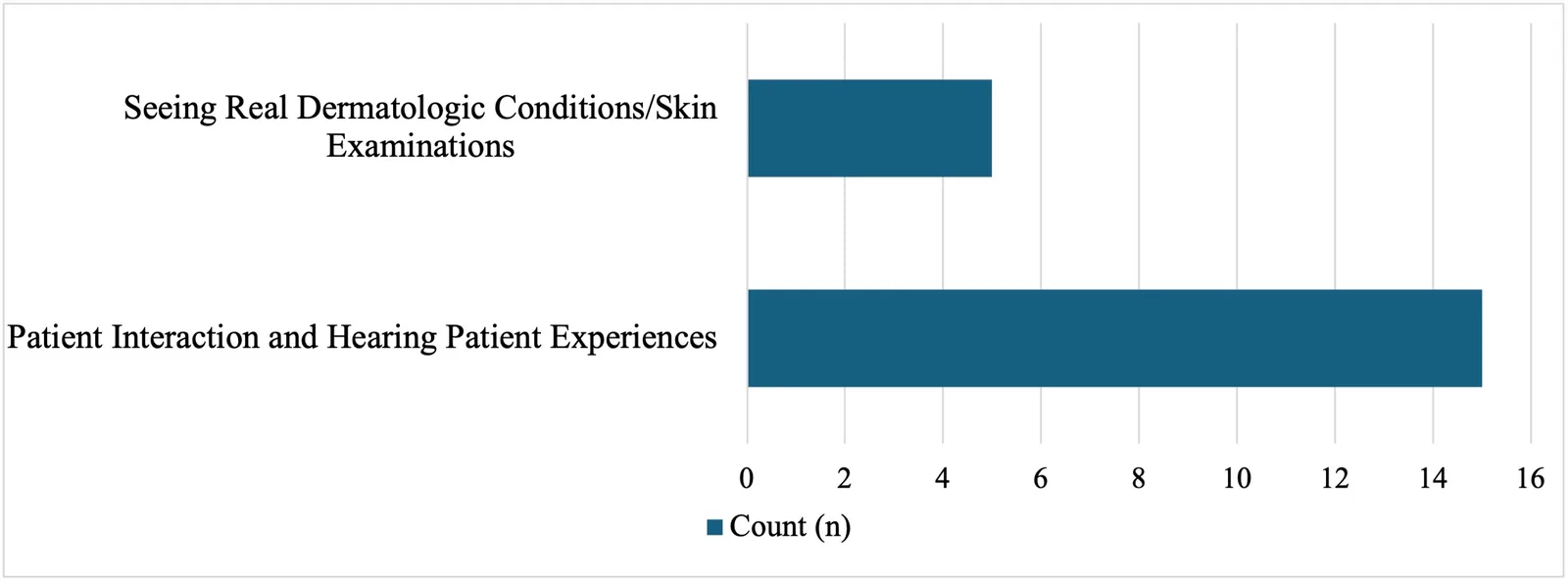
Themes identified from student responses about the most valuable component of Derm Day. Free-text responses were analyzed using inductive thematic analysis to identify recurring concepts. Each response was coded for one or more themes based on content. Bars represent the frequency with which each theme appeared across responses.

**Figure 2:**
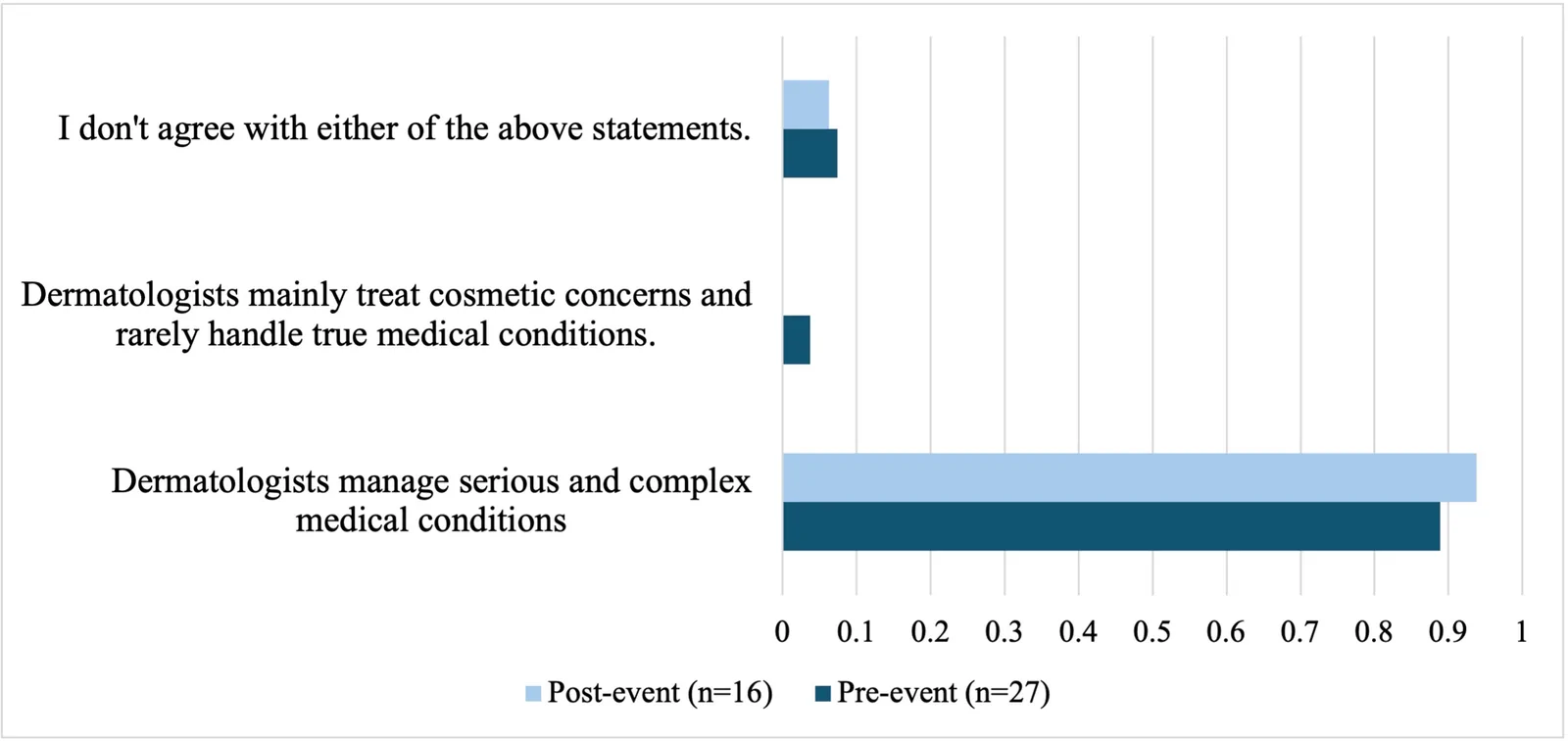
Participant responses to the statement they most agree with regarding dermatology. Bars display the percentage of students selecting each statement in the pre-event (n=27) and post-event (n=16) surveys. Responses reflect students’ perceptions of dermatology’s clinical scope.

Qualitative feedback further supported these findings. Two dominant themes emerged from free-text responses (Figure 1). First, students emphasized the educational value of interacting with patients living with chronic dermatologic conditions, noting that hearing patient perspectives enhanced their understanding of disease burden and clinical presentation. Second, students highlighted the importance of direct visual exposure to skin lesions, reporting that seeing lesions in person reinforced morphological concepts introduced during the workshop. Several participants also commented that the event broadened their perception of dermatology and corrected prior misconceptions regarding the specialty’s clinical scope (Figure 2).

## Discussion

This study demonstrates that a brief, hands-on dermatology educational intervention can meaningfully improve medical students’ confidence in performing core dermatologic assessment skills and enhance their understanding of the specialty. Students showed meaningful gains in comfort with dermatologic history-taking, lesion description, skin examination techniques, and biopsy indications.

Interestingly, knowledge quiz scores did not significantly change following the workshop. Given the modest sample size and unequal response rates between pre- and post-intervention groups, the study may have been underpowered to detect small differences in knowledge outcomes. Additionally, relatively high baseline knowledge scores on the pre-event quiz (88.43%±18.32) may have limited the potential magnitude of measurable improvement following the workshop, which likely contributed to the lack of statistically significant change (p=0.78). Future iterations of this study might benefit from utilizing more challenging, complex, or clinically nuanced assessment questions to better capture incremental advancements in student learning. In contrast, the observed improvements in task-based performance and self-reported confidence suggest that experiential learning activities may be particularly effective for developing practical dermatologic skills [12].

The significant improvements in students’ perceptions of dermatology are also noteworthy. Prior literature has highlighted that medical students often underestimate the medical complexity of dermatology and may associate the field primarily with cosmetic procedures [6]. Following participation in Derm Day, students reported greater understanding of the breadth of dermatologic disease and the role of dermatologists in diagnosing and managing serious medical conditions. These shifts in perception may be particularly important for fostering early interest in dermatology and promoting a more accurate understanding of the specialty [14]. Educational gaps in the representation of skin of color within medical curricula have also been documented, emphasizing the importance of targeted instructional efforts in this area [15].

Qualitative feedback collected from students demonstrated the value of experiential learning. Students frequently described patient interaction as the most impactful component of the workshop. Furthermore, a theme that emerged among responses was that the opportunity to directly observe dermatologic lesions reinforced morphological concepts that are often difficult to fully appreciate through textbook images alone.

Several limitations should be considered when interpreting these findings. The unequal response rates between pre- and post-intervention surveys introduce the possibility of attrition bias and response bias. Because surveys were administered anonymously and responses were not linked across timepoints, it was not possible to directly compare baseline characteristics between pre- and post-intervention respondents. The study was conducted at a single institution with a relatively small sample size, which may limit generalizability and statistical power. Additionally, the pre-post study design without a control group restricts causal inference regarding the observed improvements. The short duration of the intervention precludes assessment of long-term knowledge retention or sustained changes in specialty interest. It is also important to consider that the major findings of the present study rely on self-reported confidence, which is prone to social desirability bias and response shift bias. Furthermore, while the knowledge quiz and the five-point lesion description rubric were developed by dermatology faculty to match core curricular objectives, these instruments were not formally pilot-tested or psychometrically validated prior to implementation. Consequently, these instruments may have had limited precision in detecting small performance changes, particularly within the context of a modest sample size. Despite these limitations, the consistent improvements observed across multiple educational domains suggest that even short, structured interventions can meaningfully enhance dermatology education early in medical training.

## Conclusions

Dermatology remains underrepresented in most undergraduate medical curricula despite the high prevalence and clinical importance of skin disease. This study demonstrates that a brief, structured dermatology workshop incorporating didactic teaching, hands-on skills training, and patient interaction can significantly improve medical students’ confidence in core dermatologic assessment skills and enhance their understanding of the specialty.

These findings suggest that concise experiential learning interventions such as Derm Day may represent an effective strategy for expanding dermatology exposure within early medical education. Future studies should evaluate long-term retention of dermatologic skills, explore optimal integration of similar interventions into medical school curricula, and assess whether repeated or expanded dermatology experiences further improve students' competence and specialty understanding.

## Appendices

Survey instrument

**Table 3:** Knowledge assessment items.

Question no.	Questions	Response options
1.	Which of the following best describes a raised, fluid-filled lesion less than 1 cm in diameter?	Macule, papule, vesicle, plaque, and nodule.
2.	A patient has a pink, pearly papule with telangiectasia on the side of their nose. What is the most likely diagnosis?	Psoriasis, dry skin, acne, basal cell carcinoma, and squamous cell carcinoma.
3.	What is the first-line treatment for ringworm in an otherwise healthy patient?	Topical corticosteroids, topical anti-fungal, and topical antibiotics.
4.	Match the term to its correct description (see Matching item table).	Macule, papule, plaque, and pustule.
5.	Which of the following skin findings should raise concern for possible melanoma?	Uniformly tan lesion with regular border; symmetric brown patch unchanged for 5 years; flat red rash improved with emollients; flesh-colored papule; and asymmetric lesion with irregular border and color variation.

**Table 4:** Matching terms and description.

Terms	Description
Macule	Flat, non-palpable discoloration
Papule	Small, elevated solid lesion
Plaque	Elevated, solid lesion >1 cm
Pustule	Small, elevated lesion filled with purulent material

**Table 5:** Lesion description scoring rubric.

Criterion	Points
Primary morphology	1
Color	1
Border characteristics	1
Anatomical location	1
Secondary features (e.g., scale)	1
Total possible	5

**Table 6:** Confidence items (Likert scale 1-5). Response scale: 1 = strongly disagree; 2 = somewhat disagree; 3 = neither agree nor disagree; 4 = somewhat agree; and 5 = strongly agree.

Item no.	Statement
1.	I know what kind of questions are important to ask when a patient comes in with a dermatologic condition.
2.	I feel comfortable observing and describing skin findings.
3.	I understand the purpose and process of a full skin exam.
4.	I understand when and how a skin biopsy is performed.

**Table 7:** Perception items (Likert scale 1-5). Response scale: 1 = strongly disagree; 2 = somewhat disagree; 3 = neither agree nor disagree; 4 = somewhat agree; and 5 = strongly agree.

Item no.	Statement
1 (pre)	I understand what dermatologists do in clinical practice.
2 (pre)	Before today, I had a clear idea of what a dermatologist treats.
3 (pre)	I am aware of the challenges in diagnosing conditions on skin of color.
1 (post)	I understand what dermatologists do in clinical practice.
2 (post)	After today, I have a clear idea of what a dermatologist treats.
3 (post)	I am aware of the challenges in diagnosing conditions on skin of color.

**Table 8:** Forced-choice perception item.

Question	Response options
Please select the statement that you most agree with regarding dermatology.	Dermatologists manage serious and complex medical conditions; dermatologists mainly treat cosmetic concerns and rarely handle true medical conditions; and I don't agree with either of the above statements.

**Table 9:** Open-ended questions.

Question
What part of "Dermatology Day​​​​​​" did you find most valuable?
Is there anything you'd suggest for future "Dermatology Day​​​​​​" events?
